# Exploring Public Health Nurses’ Thoughts, Needs and Expectations for the Development and Usability of an Online Parenting Resource on Early Nutrition Delivered through Primary Care: A Qualitative Study

**DOI:** 10.3390/nu16172861

**Published:** 2024-08-27

**Authors:** Christine Helle, Elisabet Rudjord Hillesund, Nina Cecilie Øverby

**Affiliations:** Department of Nutrition and Public Health, Faculty of Health and Sport Sciences, University of Agder, 4604 Kristiansand, Norway; elisabet.r.hillesund@uia.no (E.R.H.); nina.c.overby@uia.no (N.C.Ø.)

**Keywords:** public health nurses, parental support, Infant and child nutrition, digital interventions, qualitative methods

## Abstract

Public health nurses (PHNs) constitute an important source of nutritional knowledge for parents during the child’s first 1000 days of life, but parents also seek advice from various online sources. Access to timely digital interventions may facilitate healthful eating habits later in life. In the Nutrition Now project, we wanted to combine previously evaluated digital interventions on early nutrition and implement the integrated intervention at municipality level. We prospectively explored PHNs’ thoughts, needs and expectations regarding the development and usability of such a digital resource. Semi-structured interviews were conducted with six PHNs, and data was subjected to thematic analysis. Four main themes were identified: (1) an online resource on nutrition may be a useful tool; (2) the content should attract interest and be broad in scope; (3) it must be easy to apply and adapted to different users; and (4) participating in a development process should seem meaningful. Our findings highlight the need for easily accessible, quality-assured online information to underpin the guidance provided by PHNs. The study further sheds light on prerequisites considered by PHNs to be crucial for parents to engage in a digital resource, as well as their perspectives on how it best may be communicated and used.

## 1. Introduction

The first 1000 days of life, from conception until a child’s second birthday, is a window of opportunity for promoting long-term health and well-being [1,2,3,4,5]. Exposure to a healthy diet during this period is of fundamental importance for optimal development. Failure to meet nutritional and dietary needs is strongly linked to a raised lifelong risk of obesity and non-communicable diseases (NCDs) [6], and there are also indications of links to mental health challenges [7]. In addition to nutritional needs, beneficial food and meal experiences and parental feeding practices are important for child cognitive and motoric development and the formation of healthy eating habits [8,9]. This provides parents of infants and toddlers with an important and challenging task as they are key actors in the dietary care of their child.

Public health nurses (PHNs) working in primary health care constitute an important source of knowledge and guidance for parents of infants and toddlers [10]. In Norway, all children are regularly followed up on by PHNs at municipal child health centers (CHCs) during the preschool age. The PHNs monitor the children’s development and provide guidance on early nutrition and feeding practices in line with the Norwegian official recommendations for the service [11]. According to these guidelines, topics related to nutrition are to be addressed in every consultation during the child’s first four years of life. A recent paper from our research group showed that PHNs consider the child’s diet to be an important issue for parents. Nearly all consultations at the CHCs include some discussion about diet and feeding, and the PHNs experience that they have parents’ trust when guiding on dietary issues [10]. This is in line with earlier studies from Norway and other countries, finding that PHNs consider dietary guidance to be an important topic in their consultations with parents [12,13,14], and that parents have confidence in the counselling provided by PHNs [15,16,17]. Still, parents also seek advice from other sources. In particular, parents use the internet to guide parental health-related decisions and to search for information about their child’s health-related symptoms [18]. However, finding trustworthy information on the internet may be difficult. Several actors have commercial or conflicting interests in the advice they give, making it challenging for parents to assess which are the best sources of information [19].

Access to timely digital interventions that target early nutrition and parental feeding practices may facilitate healthful eating habits later in life. Over the last 10 years, several digital resources aiming to improve child diet early in life have been developed and evaluated. A recent systematic review reporting data from 11 digital parent-supporting interventions showed that such resources can be effective in improving nutrition-related outcomes in children and parents [20]. In our research group, we have previously developed, evaluated, and reported the effectiveness of several digital resources targeting diet in pregnancy, infancy and toddlerhood, respectively [21,22,23]. In the Norwegian Nutrition Now project, we wanted to combine these interventions and implement the integrated intervention at municipality and county level [24]. To ensure that an intervention is relevant and in line with context, important stakeholders’ perspectives are essential to provide insight and ensure that the intervention meets end-users’ needs [20]. We therefore involved public health nurses in the development of the integrated intervention, as their experience provides an overall insight into parents’ needs and challenges when it comes to diet during the first 1000 days of life. In the current paper we explore PHN’s thoughts, needs and expectations for the development and usability of a new online resource on early nutrition for parents of infants and toddlers.

## 2. Materials and Methods

### 2.1. Study Design

This paper presents data collected from six semi-structured individual interviews with public health nurses working at a municipal child health center. A qualitative methodology was adopted to explore thoughts, needs and expectations for the development and usability of a new online parental resource on early nutrition designed to be delivered through municipal child health centers. The study is reported following a framework of Standards for Reporting Qualitative Research (SRQR) [25] (Table S1).

### 2.2. Setting, Participants and Recruitment

Before this study took place, there existed an agreement of intent between the managements of the university and the municipality involved in this study, intending to enhance research collaboration. One of the authors (NCØ), who is also the leader of the research team, contacted the leader of the municipality’s only child health center, who gave permission to approach and recruit PHNs to participate in the study. The CHC is located in a medium-sized Norwegian city. Purposive sampling was conducted in accordance with the leadership of the CHC. A total of 12 PHNs worked with children aged 0–5 years at the current CHC; all nurses were female. In December 2020, six of the 12 PHNs were asked to be interviewed, chosen based on variation in age and experience. All six agreed to participate in the study. The nurses were invited to the study and provided with a participant information sheet by email by the first author (CH) well in advance of the interviews. All the semi-structured interviews were conducted in January and February 2021 by the first author (CH). The recruitment process is described in more detail in a previous paper [10].

### 2.3. Data Collection

A semi structured interview schedule was developed by the authors, who have expertise in early nutrition and child health, development and evaluation of digital interventions, and qualitative methods. We used open-ended core questions, allowing the interviewer to explore issues raised by the interviewee.

The interview was divided in two distinct parts: the first part explored the PHNs thoughts and experiences related to the nutritional guidance of parents at the CHC; these results are previously reported [10]. The second part of the interview (see Table 1), intended to explore the PHNs thoughts on participating in the development process of a digital dietary tool for parents of infants and toddlers, their ideas about how such a digital resource could be fitted as best as possible to reach the end-users, and their thoughts regarding how an online resource could be incorporated in the nutritional guidance of parents at the CHC.

No relationship was established between the interviewer (CH) and the interviewee before the commencement of the interviews. All interviews were conducted at the CHC during regular working hours, with only the interviewer and interviewee present using a Zoom H1n Recorder. The consent form was signed in advance of each interview. The interview began with CH introducing herself and informing that the interview was going to be recorded, before briefly reviewing the aim of the interview. The anonymized interviews were transcribed verbatim in Norwegian by two trained research assistants. The interviews lasted on average 47 min, ranging from 33 to 54 min.

### 2.4. Data Analysis

The transcripts were uploaded in Nvivo 14 for analysis. The researchers brought in different perspectives to the analysis. Two of the authors are nutritionists (NCØ, ERH), while the first author (CH) is a child psychiatrist. As a multidisciplinary research team, we acknowledged how our individual understanding together with our academic and clinical experiences could influence the analysis.

We used an inductive, data-driven thematic analysis to explore the latent meanings and create a deeper understanding of the PHNs’ perspectives. Thematic analysis provides an accessible and theoretically flexible approach to analyzing qualitative data that can be used across a range of epistemologies and research questions [26]. It is a widely used method in qualitative health and well-being research [27], and is particularly relevant to applied research settings [28].

Through repeated readings of the transcribed interviews, a comprehensive understanding was achieved, and quotes were selected. Through continuous revisions, a common understanding and agreement on themes and subthemes across the research team was achieved. Data saturation was reached, as no further new information was provided in the last interview. The analysis used a prescribed “step-by-step” process following Braun and Clarke’s guidelines of thematic analysis [26], presented in Table 2.

### 2.5. Ethical Considerations

The present study is part of the Nutrition Now project. The Norwegian Centre for Research Data evaluated and approved the study (26 March 2020, Reference 137889), the study was conducted in accordance with the Declaration of Helsinki.

## 3. Results

Semi-structured interviews were conducted with six out of twelve nurses; all nurses were female. The number of years working as a PHN varied between 3 and 40 years, but all the interviewees had more than 10 years of experience working as a nurse. The current CHC covered the entire municipality, and all PHNs worked with families from both high and low socioeconomic areas. One of the PHNs had a special responsibility for working with refugee-families, though all nurses regularly followed up immigrant-families.

**Figure 1:**
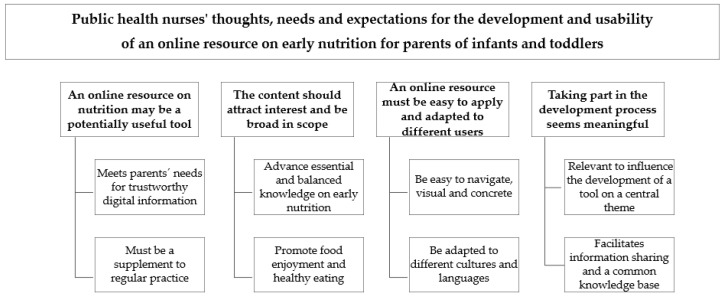
Themes and sub-themes identified during thematic analysis.

Across all the interviews, four thematic areas and eight sub-themes were identified; see Figure 1. The thematic areas and their respective sub-themes are summarized below with illustrative quotes. A table with all the quotes that formed the basis of this analysis is included in the Supporting Information (Table S2; Quotes).

### 3.1. Theme I: An Online Resource on Nutrition May Be a Potentially Useful Tool

It became clear from the interviews that the PHNs experienced a need for safe and quality-assured online resources that could be communicated to parents. However, the thematic analysis also revealed that the PHNs had some objections regarding use in consultations, as presented in the following two sub-themes.

#### 3.1.1. Meets Parents’ Needs for Trustworthy Digital Information

Although some PHNs were concerned about excessive use of screen time among parents, all the PHNs acknowledged the internet as parents’ first choice when searching for information. The PHNs reported that parents use the internet extensively to search for information and that they often “google” to access advice on issues of concern. This was also consistent with how health information provided at the CHC had shifted from use of paper brochures to web-based information.

… it’s a bit like that, we’re also very concerned to talk about limiting screen use and all that, so there’s always a discussion about whether we refer too much to websites and the like. But, in a way, that’s where people are, so …

(Interviewee 1)

Yes, the digital things we have are there to support parents and it is nice that it is digital rather than on paper …

(Interviewee 3)

We just have to realize that this is where people get most of their information now. And then it is much more important that they have some good sites, good websites where they can find information, than all the other websites that they also search for …

(Interviewee 6)

The professional quality of the websites that parents used to search for information varied considerably. Although the PHNs reported that some parents used official Norwegian webpages, they very often experienced that parents referred to different blog-forums or the like. The PHNs expressed that it would be helpful in their practice if they could refer parents to a safe and quality assured parental website on early nutrition.

No, it’s very varied … some people are a bit aware, they use the Norwegian Directorate of Health and such sites, but there are many parents who use such … blog things and the like, and … I think it varies a lot. (…) Yes, in any case it is better to have safe sides to … show then … yes.

(Interviewee 1)

And that they google a lot themselves, that’s for sure, because sometimes it happens that they come here and we talk about things, and then they’ve googled their way to something that is … far from what I can vouch for, and then we can talk a bit about that, right …

(Interviewee 4)

Although the PHNs were generally positive about an online resource on early nutrition, they did not believe that this was something that all parents would use. This particularly applied to parents with lower socio-economic status.

… I don’t know what research says about online information? I would think that there are … socio-economic differences in what people do, like those with higher education exercise more and have a better diet. And maybe it will always be like that, but … in any case, you can’t give up trying to make parents aware. So, I think that, yes, maybe there may be differences, but it’s still good to have then. And then it’s up to us nurses to catch this and guide further if someone … isn’t there … I think. (…) But … I feel that … I think it will be a useful tool to have … for everyone, regardless of background.

(Interviewee 2)

… it is probably those who come out worst in many other contexts as well. Those who, in a way, are not used to searching, perhaps do not have much education themselves or are used to looking for information about what they are wondering about. Yes, you may say, those who are a bit left out in many ways then.

(Interviewee 5)

#### 3.1.2. Must Be a Supplement to Regular Practice

Throughout the interviews, it became evident that both personal contact and face-to-face dialogue were highly valued by the PHNs. Although they responded positively to an online tool on early nutrition, they were more skeptical about using it directly in consultations with parents. The PHNs feared that the screen could steal focus from the conversation and personal guidance, which had to come first.

I’m not a big fan of us having a screen during the consultation, so if that’s how it’s set up, I personally don’t think it’s that nice to work like that and have a screen up.

(Interviewee 3)

No, in my experience, showing films requires a lot of concentration, when we have to scroll up the screen. And if you are going to have more films to show … if that is a part of the program then …

(Interviewee 3)

So, it is clear that this will be in addition to … Because I think that the personal contact, the conversation we have, that is the most important thing (…) That this will be a supplement, you can’t just say that “you can read about diet there” in a way. That would be too simple.

(Interviewee 6)

On the other hand, the PHNs expressed that a website on early nutrition could yield good support and be a useful tool if added to, without replacing, their usual practice. A digital resource on early nutrition could be something the PHNs may refer to for parents to watch at home, either before or after the consultation, reinforcing the content of the conversations at the CHC.

Yes, I think you have to take the … guidance first, and then in a way have it (the digital resource) as a … it might be a bit of extra information then … in the same way as showing them a brochure, «go in and look at this”. Because there’s not always a need for you to look at it together/…/yes … and so that it will be, in a way, a supplement to the conversation and the guidance we otherwise have.

(Interviewee 1)

No, it has to be something that we can, in a way, use in addition to what we have on the program, and which we are communicating about.

(Interviewee 4)

Some of the PHNs said that an online resource could act as a neutral disseminator of information on sensitive subjects, such as overweight. Conversations about overweight or obesity were perceived by many as difficult and could sometimes arouse aggression when brought up.

And where it is vulnerability, some might get a little angry when we talk about obesity issues … The way I see it, it might be a support for us … because then such a digital platform would be very neutral, whereas I would not be so neutral. So, then I could support the neutral information, I could build on that then, if I gave up the personal point of view, but referred to it, then maybe that would support the guidance.

(Interviewee 3)

Among the PHNs there was also some resistance to including new procedures in their consultations, as there were already many topics to be covered. If they were to bring in something new, it had to be based on their own assessment of relevance.

And there is so much we have to talk about, and sometimes there are some really difficult issues … breakups and so on, there is so much that may come up like that. So, if it’s like “oh, I have to get that film in as well”, it may be a bit … But then I think that, if necessary, if I see the need … (…) But getting it in to everyone, I think that can be difficult.

(Interviewee 5)

### 3.2. Theme II: The Content Should Attract Interest and Be Broad in Scope

#### 3.2.1. Advance Essential and Balanced Knowledge on Early Nutrition

The PHNs declared that in an online resource on early nutrition, it is important to communicate what constitutes a healthy diet early in life in a simple and clear way. The information provided should be unambiguous, while allowing for individual adaptations without arousing feelings of guilt or shame.

In any case, there should be no room for misunderstandings. It is important to think about that. Maybe the use of language, it cannot be offensive … I don’t think that there are … that many things that aren’t okay, really (…) but that there is some room for individual differences … That it’s not one answer to what to eat, there may be room for like … when the child doesn’t like something, he can try something else, right …

(Interview 2)

So, in some ways you have to find a balance where … at least try to see what is good enough, and not give them worse conscience for everything that they can’t afford, manage or bear nd so on …

(Interviewee 4)

Some of the PHNs pointed out the challenging task of creating a digital resource on early nutrition that is suitable for everyone and captures the interest of parents who may not be interested.

… and find a way that reach parents regardless of competence, dyslexia, language … and the educational level of the parents. (…) That it should be suited for teenage parents as well as new, anxious parent at 40 years of age (…) It should somehow reach all without making them think that this is too simple or basic, and at the same time fit a 19-year-old.

(Interviewee 3)

Yes … they must in a way … want it and be interested and … some, we see that with the other websites we recommend … some are very keen, and others are just not interested. And some parents have an easier time figuring it out than others, so that (…) While maybe … those who doesn’t have many thoughts about it in a way, they just google it and then they find an answer, so these are the ones you in a way would like to reach then …

(Interviewee 5)

The PHNs reported that they often receive questions on nutrition during the child’s first year, especially related to the infant’s introduction to solid food.

Eh … so, there are often questions that you have to figure out, that you haven’t thought about and such, but … there are a lot of questions anyway … the first, like when they start with food then, solid food. And… which food to serve the child?

(Interviewee 1)

Yes, that assessment right here and now for each child when the nutrition is still breast milk, when is it right for my particular child to start with solid food in addition to milk? (…) And yes, I would like it to have information on that. Help and support for this part …

(Interviewee 3)

Other questions could deal with the amount of food to be eaten, the need for various nutrients, and nutritional content in different food types.

Yes, maybe a bit of that … I feel that many people want the best for their children, but then they make mistakes because they don’t know, for example, how many calories there are in … various products then … i.e., everything that has a bit of hidden calories… like e.g., dairy butter or whole milk or yoghurt… ready-made food. The food that they really think it’s healthy, but it’s not… (…) And the amount of food … how much food should a child have? Because some parents are satisfied if the child has eaten a bunch of slices of bread, but you don’t need all that when you are two years old … so the amount of food and, it might be a good idea … to inform parents about that.

(Interviewee 2)

… then I think about having enough iron. A diet rich in iron then … so not additives, but what is found naturally in food, where to find it most easily and how important it is … And the combination of calcium and vitamin D …

(Interviewee 4)

The majority of the PHNs expressed that guidance on allergies and different diets could be challenging, as questions on these topics had increased in scope and frequency. Sometimes they felt that they fell short in these subjects.

I don’t know if it is included, but this with vegetarian or alternative diets. Or diets in general … taking away foods that the parents think the child can’t tolerate—we see it a lot. (…) Yes, too often the parents have a very … they are very determined that “My child can’t eat this and that”, and it is really just nonsense.

(Interviewee 5)

It is often the case that many parents have thoughts that their child cannot tolerate this or that. And there is no professional basis for saying so, but nevertheless they have made up their minds. So I think it’s also useful to know a bit about such things. But there’s also something about not focusing too much on it, because then there might be even more people thinking about it … but it’s something I’m seeing more of now … intolerant to milk, intolerant to gluten or yes …

(Interviewee 6)

#### 3.2.2. Promote Food Enjoyment and Healthy Eating

Promoting a healthy and positive relationship with food and meals from an early age was important to the PHNs. They experienced that parents struggle both when children are picky and when they eat too much, and that these struggles could influence food and meal enjoyment over time. An online resource could give parents advice and tips, while confirming that they were not alone in their struggles.

Yes, I think that then … there are those who struggle, if the child is not fond of bread, for example, or you have a type of genetic variant, a bit of a difficult, picky eater type. There are a lot of children who, I see that often either the mother or father may have been like that as a child, right, and then it gets better around 6–7 years of age and then … maybe have a resource for this? (…) I think it would be quite … nice for the parents to see that “it’s not just my child who is like that, this is a known problem”.

(Interviewee 2)

No, but what I think many parents can struggle with is that the children don’t like the food, don’t want to taste it and things like that. And they can easily fall into the trap of offering something else instead. So I think it can be very useful for many to get advice, help and guidance on such issues. And provide support to have the strength and courage to stand in situations where the child does not want or wish to taste”.

(Interviewee 6)

Early food experiences with a focus on sensing and learning were perceived as important, as was the interaction between child and parent during mealtimes.

… something that I think could be useful for the users? It’s more support in the … what should I say, the sensing of food. Not just physically getting food into the stomach, but sensing, learning about food, oral motor skills, enjoying the meal and the food. That this has to be a very big part of the package, that it’s not just about which nutrients and which types of food (…) The early introduction of food, the sensing and the positive approach to nutrition …

(Interviewee 3)

Yes, yes, it’s a big topic. And maybe a little bit about this … who’s in charge here? You’re the adult and you decide what goes on the table and what’s available in the cupboard (…) And the interplay between food and emotions, to use a soon-to-be worn-out expression. But it’s so intertwined, so making it positive – that it (the resource) can help us to focus on it together with the parents.

(Interviewee 5)

### 3.3. Theme III: An Online Resource Must Be Easy to Use and Adapted to Different Users

The PHNs were concerned that an online resource on early nutrition should be developed specifically with the aim of reaching those who had little interest in the topic and were difficult to reach with ordinary guidance. Their thoughts on necessary prerequisites for reaching these users are presented in the following sub-themes.

#### 3.3.1. Be Easy to Navigate, Visual and Concrete

For a digital resource to be used, it had to be adapted for a smartphone. While the PHNs were uncertain whether everyone had access to a PC, they claimed that all their users had their own smartphone.

Yes, everyone has it now. Maybe not a PC at home, but smartphones have … almost 99.9% must have it, that’s my impression. Because here you get a text message the day before “remember you have an appointment for little …” or … yes.

(Interviewee 2)

Oh yes, everyone has (…) It … I’ve hardly met any families who don’t have a smartphone. (…) No, they’re very keen on it. Both to be able to Skype or have some Facetime conversations with family in their home country and … no, so everyone has one.

(Interviewee 4)

The PHNs believed that an important potential barrier to use would be whether the digital resource was easy to access and navigate.

… but it must be easy to find your way around and so on … simple in that sense. Yes …

(Interviewee 1)

It must at least be easy to find and said in a very simple way.

(Interviewee 5)

Further, a digital resource on early nutrition must present the most important content easily on sight. The text should be short and concise, and the information can beneficially be provided in a visual form such as videos or pictures.

And those who have few resources, if there’s a lot to read, they may drop out. (…) At least according to feedback on other things we’ve had over the years, things with a lot of text, people don’t bother with that, but a bit like that … point by point (…) Yes, I think at least in order to have an effect on living conditions and such, that it has to be short and concise, so they can delve deeper if they want to, but I think many people drop out if you have to read to the bottom before getting the clue.

(Interviewee 3)

It’s certainly important that the information is easy to find and said in a very simple way, and maybe a bit like … So that it’s not too boring, that it’s a bit exciting, I was going to say. (…) Yes, make it short.

(Interviewee 5)

#### 3.3.2. Be Adapted to Different Cultures and Languages

Language could be an important barrier to good communication. All the PHNs had experienced that parents with a mother tongue other than Norwegian more often failed to understand the information given. For a digital resource on early nutrition to be perceived as relevant for all users, it had to be adapted to different languages and cultures.

Yes, it would be nice if it were in many languages. Because it’s often very challenging, especially perhaps those who … know a little Norwegian … who you might not have an interpreter for, because you think that this is fine, right … and then they might not want it either. And I’ve experienced that many times, when they come back … you think they understand, and then they don’t. (…)

(Interviewee 1)

Well … it depends on it being in a familiar language … because … It takes a long time before they can master that type of information (…) because there are details and nuances that are not … when you have to explain things or talk about things that … are a little deeper than simple “yes and no” and … yes … so it has to be … available in other languages.

(Interviewee 4)

### 3.4. Theme IV: Taking Part in the Development Process of an Online Resource on Early Nutrition Seems Meaningful

#### 3.4.1. Relevant to Influence the Development of a Tool on a Central Theme

Although there were many different projects to be involved in at the CHC, the PHNs were positive about participating in the process of developing an online parental resource on early nutrition. Nutrition was confirmed as an important topic at the child health center, brought up in almost every consultation during the preschool-years. Promoting a healthy diet from an early age was seen as an important primary preventive measure, and taking part in such a process could give the PHNs greater ownership of the project.

I think it’s good … yes … I’m in favor of it (…) I think so. Because food for those children is a recurring topic we often talk about”.

(Interviewee 2)

So, diet is linked to many lifestyle diseases. In terms of prevention, it’s very important (…) We’re in a period of a lot of projects right now, and that’s been my little concern in all of this (…) Because we feel like we’re being inundated with things that we have to use and communicate and incorporate all the way. But if it’s something short-term that we’re involved in, and if it results in good material that we can use, then I think there’s interest in that …

(Interviewee 5)

#### 3.4.2. Facilitates Information Sharing and a Common Knowledge Base

Participating in the process of developing an online resource could raise an opportunity for gaining new knowledge and experience on a topic that was essential at the child health center.

No, I think it can help to raise the importance of nutrition.

(Interviewee 5)

I think it’s very exciting because we get to raise these topics again, and we get a bit of the latest research and things like that. And a bit more interdisciplinary in a way, so you learn more, which is very exciting. It’s definitely positive for the child health centerto be involved in something like that (…) Yes, I think we’re generally very interested in gaining more knowledge. I definitely think so.

(Interviewee 6)

Participating in the project could further contribute to greater consensus and coherence among the public health nurses. Even though they worked very independently, having individual consultations, the PHNs emphasized the importance of having a uniform practice regarding giving advice and guidance.

… I haven’t worked that long, others have worked for more than 30 years. But things change … all the time, and then there’s something about us … that we all give the same advice … because it’s a bit like that … we sit in our own offices and … yes, it’s important … that we don’t give advice in different ways, even though it has to be adapted to each individual family …

(Interviewee 1)

… and that’s how it is with all topics, to always … aligning ourselves and being on the same level. Maybe it’s easier to (…) Yeah, I don’t know. Quality assurance for users, because we’re very concerned about that. That what we deliver must have the same weight, or be at the same level. And that it should be the same, that it doesn’t matter if they come to see me or her.

(Interviewee 3)

## 4. Discussion

The current study prospectively explores public health nurses’ thoughts, needs and expectations for the development and usability of an online parental resource on early nutrition to be delivered by PHNs in primary health care service. Our main findings highlight the need for easily accessible and quality-assured web-based information on early nutrition to support and underpin the personalized nutritional guidance provided by PHNs. The current study further sheds light on assumptions considered by PHNs to be crucial for parents to engage in such a digital resource, as well as their perspectives on how an online resource can be communicated and used at the child health center in the best possible way.

### 4.1. A Need for Secure, Digital Information as a Supplement to Regular Practice

All the PHNs said that the internet is parents’ first choice when searching for information. They did, however, frequently experience that parents referred to webpages and blog sites of varying quality. This is in line with findings from other studies exploring parental use of health-related digital information. A systematic review from 2020 found that parents are heavy users of health-related information on the internet across highly diverse circumstances [18]. For the most recent studies, the Google search engine was used by almost all parents as a starting point. Other studies confirm that parents do not routinely use websites that are known to provide safe, accurate, and reliable information [19], and they strive to assess the quality and reliability of the websites they are using [29]. Taken together, our findings suggest that in addition to providing personalized nutrition counselling to parents at the CHC, PHNs also need to guide parents on where to find reliable online information. One possible way to address this need could be to introduce parents to and familiarize parents with a shared and tailored digital dietary tool at the CHC. In the Swedish Ministop trial, Alexandrou et al. recently developed and evaluated a culturally adapted version of their parental support app promoting healthy diet and physical activity [30]. They reported that nurses in primary child health care underlined the importance of a shared digital resource between parents and themselves to facilitate communication. Our findings underpin the importance of primary care providers supporting parents in their digital information seeking and support the potential benefits of a shared digital tool on early nutrition that can be incorporated into routine practice.

Although the PHNs uttered a wish for “a safe digital tool” in line with national recommendations to support their nutritional guidance, they were more skeptical about using this within their consultations. An online resource should preferably be used flexibly and when needed, as a supplement to regular practice, not as an enforced routine. One of the reasons given was that the PHNs feared that screen use would come at the expense of conversation and personal contact. We have previously reported that PHNs place great emphasis on building mutual confidence and trusting relationships with children and their parents [10]. An established relationship enables the PHNs to raise personal issues related to diet and eating behaviors and provide nutritional guidance in a more customized way. The concern that screen use can negatively impact the patient relationship is supported by research. A systematic review exploring patient-related communication in the era of electronic health records reported that this may interfere with collection of psychosocial and emotional information and therefore interfere with development of supportive, healing relationships [31]. Our findings suggest that if digital interventions are to be carried out by public health nurses at child health centers, it is important that enough time is set aside to ensure that this does not compromise personal contact and individually tailored guidance.

Another reason for the PHNs’ skepticism about routinely displaying information on a screen was lack of time. The official Norwegian recommendations for child health service [11] imposes many different tasks on the PHNs, and time constraints make prioritization important. If something new was to be included in the consultations, something else had to be excluded. This could be an obstacle to implementing a digital tool as a fixed routine. A recent paper by Toomey et al. explored healthcare professionals’ views on the acceptability of delivering interventions to promote healthy infant feeding practices within primary care [32]. They found lack of time and too many competing priorities within the existing healthcare professional role to be important barriers to delivering infant feeding interventions. It was essential that interventions were developed in collaboration not only with parents, but also with the stakeholders who would be involved in rolling out and implementing the interventions, and that relevant system-level actors like policymakers ensured sufficient resourcing.

Lastly, the PHNs expressed concern about screen use among parents of infants and toddlers, and some PHNs were ambivalent about recommending new websites to parents on a regular basis. This concern is not unfounded, as studies point to a relatively high prevalence of parental occupation with digital devices while interacting with their children [33,34]. Many everyday disruptions to parent–child interactions, such as frequent use of a smartphone, are likely to decrease parenting quality [35] and might also affect optimal development of the child’s early social, emotional and cognitive skills [36]. These negative consequences of engaging in screen use also apply to mealtimes. Mealtime screen use has been associated with both poorer dietary outcomes in children [37] and reduced opportunities for family cohesion at mealtimes [38]. A qualitative Australian study found that although family mealtime screen use is linked with a range of child behaviours and parenting practices that may negatively influence children’s dietary intake and social engagement, parents often considered screens acceptable at mealtimes [39]. Today’s parents are using digital devices extensively and the healthcare sector is increasingly using the internet to communicate patient-centered information [40]. A challenging task for PHNs may therefore be to guide parents on the use of recommended and informative websites, while at the same time making parents aware of the potential negative impact frequent use of a smartphone or tablet may have on infant–parent interplay and mealtimes. In today’s era of digital devices, it is important to give PHNs sufficient time and resources to guide parents on screen use related to daily care tasks. A shared digital resource on early nutrition, addressing the benefits of screen-free family meals, could be useful in this work.

### 4.2. The Content of an Online Resource on Early Nutrition Must Attract Interest and Be Broad in Scope

Our findings further point to the need for early nutritional guidance to be broad and comprehensive, covering a wide range of aspects related to food and meals in early childhood. According to the PHNs, a digital resource on early nutrition should encompass basic, balanced and easily accessible information on topics such as when to introduce solid food for the individual infant, the child’s need for different nutrients, food allergies and intolerances, what to do when a child is picky and how to lay the foundations for a healthy relationship with food and meals from an early age. Also, as parents vary in skills and demands, the information given should preferably balance between being clear and distinct on the one hand and making room for variation and individual considerations on the other. This may seem like a difficult balancing act; still, Norwegian public health nurses are obliged by official guidelines to provide nutritional counselling on a wide range of topics, tailored to each individual child and family [41]. Also, previous studies have shown that it is important to deliver feeding information in a way that is experienced as relevant and meaningful to different parents. A qualitative study exploring mothers’ experiences and perceptions of complementary feeding recommendations in primary care settings found that most mothers reported that some infant feeding recommendations were difficult to follow when perceived as unclear, not tailored to the child or not culturally sensitive [42]. Another qualitative study evaluating factors influencing parents’ engagement with a nutritional mHealth program found that engagement fluctuated depending on need and the degree to which the program fit with existing beliefs and values [43]. A systematic review evaluating parents’ experiences with digital nutrition promotion websites and apps [20] reported that parents wanted specific, age-relevant and tailored or personalised content and disliked general or vague information. Parents further desired practical information that supported behaviour change by, e.g., addressing barriers, and disliked content and terminology which elicited negative emotions such as fear, guilt or shame. Lastly, parents wanted information that was trustworthy, evidence-based and sourced from universities or government organisations. Our results support previous research pointing out that early nutritional guidance must be sensitive to the significant constraints and practicalities parents are faced with [44]. For an intervention on early nutrition to succeed, it is important to incorporate respect for parents’ wishes and perceptions of how they understand their child, coupled with broad, clear and trustworthy information.

### 4.3. An Online Resource Must Be Easy to Use, Concrete, Visual and Adapted to Different Languages

The current study reveals prerequisites that PHNs consider to be crucial for a digital resource on early nutrition to be feasible and easily accessible to parents. First, a digital resource must be customized for use on a smartphone. Second, the most important information should be made easily accessible to avoid the user having to take several steps to access to the desired content. Third, text needs to be short and concrete and beneficially provided in a visual form such as videos or pictures. Lastly, it should be adapted to different users in terms of language and culture. These aspects were considered as particularly important to approach hard-to-reach parents, especially parents who were foreign-language speaking or parents with a lower socio-economic status.

Health literacy, commonly understood as knowledge, motivation, and competence to assess, understand and apply health information, is an important mediator of income-, race- or ethnicity-associated health disparities [40]. Low parental health literacy has, in several studies, been linked to poor health knowledge and child health status [45]. Today, health care providers increasingly use digital technologies to communicate information to address health needs and deliver health care interventions. Still, there has been limited research investigating how parents with low health literacy use these technologies. A study from the United States (2020) reported that although using the internet to find health information was a common practice across parental health literacy levels, parents with low health literacy were less likely to use the internet and cell phone apps for health management than those with higher health literacy. The authors concluded that educational strategies that expose families to these technologies and efforts to improve the navigability of these tools would likely be beneficial for parents across all health literacy levels, but would especially benefit those with low health literacy [40]. A shared online resource on early nutrition, introduced to parents at the CHC to be further explored at home, may thus be an initiative that promotes digital health literacy as well as helps to level out social inequalities in health.

### 4.4. To Participate in the Development Process May Increase Professional Consensus and Ownership

Overall, the PHNs were open and positive to participating in the development process of a new digital resource on early nutrition. This could raise an opportunity for gaining new knowledge and experience, as well as contribute to a greater consensus among the nurses on topics they considered important. We have previously reported that even though nutritional counselling stands out as an important, multifaceted, and as a central part of the PHNs’ work at the CHC, this seems to be reflected to a lesser extent in their own education and course offerings. [10]. Also, studies from other European countries have reported that PHNs experience a limited focus on nutrition and infant feeding within existing practice nurse training [32,46]. The PHNs openness to learning and trying new tools as well as the importance of all PHNs possessing the same knowledge in order to ensure that all parents receive the same nutritional guidance, was also reported in the Swedish Ministop trial [47]. They also found, in line with our previous study [10], that the PHNs conveyed a strong professional identity and responsibility to guide parents, and considered these to be characteristics that could potentially both promote and inhibit implementation. Our findings also suggest that although the PHNs are positive regarding a new digital resource on early nutrition, it is important to ensure that the PHNs can vouch for the quality of the resource and that it can be incorporated into daily work in a satisfactory way for the PHNs to develop ownership of the resource.

### 4.5. Strengths and Limitations

This study has several strengths. The public health nurses interviewed had different experiences before becoming PHNs and had worked as PHNs for varying lengths of time, which provides different perspectives and insights. The interviewer was skilled in dialogues and experienced in early childhood care, and the co-authors were nutritionists with extensive clinical and research experience, providing broader perspectives to the interpretation of the findings.

However, there are limitations to consider. The existing agreement of intent between the university and the current municipality may have influenced the individual nurse’s consent to participate in the study, but it is less likely that this had any significance for what was communicated in the individual interviews and the results as such. Although the PHNs worked quite independently and were responsible for their own clients, they worked in the same unit which may have led to a homogeneity in their reflections and responses. Another limitation is that although the leader was asked to select the interviewees based on variation in age and experience, there may have been some subjectivity and convenience associated with the selection, which means that we cannot exclude selection bias. In addition, the low number of respondents is an important limitation of the study. The authors’ professional background may have led to confirmation bias and an increased reported nutritional focus. Lastly, our findings may not be generalizable to other regions. Although the activities of the Norwegian municipal health centers are anchored in a national guideline, there may be differences due to local conditions and our findings may therefore not be representative for the whole country.

## 5. Conclusions

This study provides valuable insight into public health nurses’ thoughts on various aspects of developing and adopting a digital parenting resource on early nutrition delivered through the primary healthcare service. Our findings highlight the need for easily accessible, quality-assured online information on early nutrition to underpin the personalized guidance provided by PHNs. Our findings also point to the importance of ensuring that PHNs can vouch for the quality of the resource and that it can be incorporated into daily work in a satisfactory manner. If digital interventions are to be carried out by public health nurses at child health centers, it is important that enough time is set aside to ensure that this does not compromise personal contact and individually tailored guidance. The low number of respondents is a limitation of the study.

## Figures and Tables

**Table 1:** Interview questions.

Can you tell me about your own background and how long you have worked as a public health nurse?
What are your thoughts on using an online resource on early nutrition in your nutritional guidance of parents at the child health center?
In what way could an online resource on early nutrition be of help to parents of infants and toddlers?
Could there be any challenges or disadvantages in introducing an online parental resource on diet and nutrition for infants and toddlers?
Are there one or more topics that you wish an online resource on early nutrition should cover?
Do you have any thoughts on what the health center might achieve by participating in this project?

**Table 2:** Phases of thematic analysis.

The Application of Step-by-Step Thematic Analysis Based on Braun and Clarke, 2006	
1. Familiarizing yourself with your data	The digital interview recordings were transcribed verbatim by two trained research assistants. All three authors (CH, ERH, NCØ) read and re-read the transcripts to become familiar with the data, noting down initial ideas.
2. Generating initial codes	Initial codes were generated systematically across the entire data set (CH), collating data relevant to each code.
3. Searching for themes	Codes were collated into potential themes and subthemes (CH), gathering all data relevant to each potential theme.
4. Reviewing themes	The authors (CH, ERH, NCØ) engaged in discussions of the themes and subthemes to achieve a shared understanding of each theme and to ensure that the themes were anchored in the related coded extracts and the entire dataset. Themes were reworked and subsequently validated across the dataset. Finally, a thematic map was generated (CH).
5. Defining and naming themes	Themes were defined (CH); co-authors (ERH, NCØ) were involved in ensuring themes appropriately reflected transcribed interviews. The introduction (NCØ, CH) and the overall story (CH) were drafted.
6. Producing the report	Finally, all themes, codes and illustrative extracts were compiled together and refined by the first author (CH) and reviewed by the co-authors (ERH, NCØ), linking the findings to previous literature and considering the broader impact of the findings.

## Data Availability

The original contributions presented in the study are included in the article; further inquiries can be directed to the corresponding author.

## Supplementary Materials

The following supporting information can be downloaded at: https://www.mdpi.com/article/10.3390/nu16172861/s1, Table S1: Consolidated Criteria for Reporting Qualitative research (COREQ). Table S2: Quotes
