# Supplementary material for: Exploring Public Health Nurses’ Thoughts, Needs and Expectations for the Development and Usability of an Online Parenting Resource on Early Nutrition Delivered through Primary Care: A Qualitative Study
Source: Nutrients. 2024 Aug 27;16(17):2861. doi: 10.3390/nu16172861 (PMC11397169; doi:10.3390/nu16172861)
Supplement: Supplementary file 1 [file nutrients-16-02861-s001.zip › Supplementary files/Table S2; Quotes.pdf]

Table S2; Quotes

Theme I	Subthemes	Sample quotes
**An online resource on early nutrition may be a potentially useful tool**	***Meet parents' needs for trustworthy digital information***	*"Today´s parents are digital"*<br><br>*Yes, I think it's... positive, because otherwise they google everything and find theirs ... so it's better to be able to point to some concrete tools that are, in a way, ..., we do that with other things ... (Interviewee 1)*<br><br>*... it's a bit like that, we're also very concerned to talk about limiting screen use and all that, so there's always a discussion about whether we refer too much to websites and the like. But, in a way, that's where people are, so... (Interviewee 1)*<br><br>*... because there is already a lot that is referred to online and such ... but we used to hand out a hundreds of brochures, and we don't do that anymore. And that... I think it's nice to have something to show when they ask. Something they can... yes, read on. (Interviewee 1)*<br><br>*Yes, I think that would have been nice. They're digital now...so that's what they want. I always try to say that they should seek research-based information, and no other things... to get the best information about everything they wonder about... (Interviewee 2)*<br><br>*Yes, the digital things we have are there to support the parents and it is nice that it is digital rather than on paper... (Interviewee 3)*<br><br>*It's nice to be able to refer to some reliable, good sources about food, because as I said ... I´ve experienced that "Oh, where should I have recommended that they looked for that?" I felt I didn't have anything immediately at hand. Sometimes I miss that. Yes, it is probably most about having something to offer the parents (Interviewee 5)*<br><br>*We just have to realize that this is where people get most of their information now. And then it is much more important that they have some good sites, good websites where they can find information, than all the other websites that they also search for... (Interviewee 6)*
		"Quality of websites varies"

		No, it's very varied … some people are a bit aware, they use the Norwegian Directorate of Health and such sites, but there are many parents who use such... blog things and the like, and ...I think it varies a lot. (...) Yes, in any case it is better to have safe sides to... show then… yes (Interviewee 1)<br><br>*I think it can be a very good support to know that here they will find safe, good and correct information and weed out other nonsense from the Internet. Because there is a lot of weird stuff that is read, and then they know that here it is safe, good and correct... (…) And a good, safe platform is needed in the throng of bloggers, yes... (Interviewee 3)*<br><br>*And that they google a lot themselves, that's for sure, because sometimes it happens that they come here and we talk about things, and then they've googled their way to something that is... far from what I can vouch for, and then we can talk a bit about that, right ... (Interviewee 4)*<br><br>*There are many people who have different types of forums from which they get information, so it is much better that we can point to places where we think that the information is safe and good. (Interviewee 6)*
		"Not all parents will use a digital resource"<br><br>*… I don't know what research says about online information? I would think that there are ... socio-economic differences in what people do, like those with higher education exercise more and have a better diet. And maybe it will always be like that, but ... in any case, you can't give up trying to make parents aware. So, I think that, yes, maybe there may be differences, but it's still good to have then. And then it's up to us nurses to catch this and guide further if someone ... isn't there ... I think. (...) But ... I feel that ... I think it will be a useful tool to have ... for everyone, regardless of background. (Interviewee 2)*<br><br>*Those who are very low functioning would probably not use it (a digital resource) at all, because then it holds in a way. They think it's more than enough, but in the main I think it will just be a support. (Interviewee 3)*<br><br>*... it is probably those who come out worst in many other contexts as well. Those who, in a way, are not used to searching, perhaps do not have much education themselves or are used to looking for information about what they are wondering about. Yes, you may say, those who are a bit left out in many ways then. (Interviewee 5)*<br><br>*... but it is clear that not everyone thinks this is easily accessible, they find it difficult to deal with this type of information. So, for some it will certainly be a bit difficult. (Interviewee 6)*

	Must be a supplement to regular practice	*"The personal contact is most important"*<br><br>*No, I think that it is positive, but there is something about not everything... that you must not lose that ... the guidance you have from before, and that relationship and contact there then ... (Interviewee 1)*<br><br>*I'm not a big fan of us having a screen during the consultation, so if that's how it's set up, I personally don't think it's that nice to work like that and have a screen up (Interviewee 3)*<br><br>*No, so the experience of showing films is that it requires a lot of concentration, when we have to scroll up the screen. And if you are going to have more films to show ... if that is a part of the program then... (Interviewee 3)*<br><br>*As for using more film in the consultation, I look a bit negatively on that because it is already so full. (...) I probably think a bit like … I think it can be difficult to use it (digital platform) in the consultation, because it becomes like "oh, shall we sit here and watch a film?". (Interviewee 5)*<br><br>*So, it is clear that this will be in addition to ... Because I think that the personal contact, the conversation we have, that is the most important thing (...) That this will be a supplement, you can't just say that "you can read about diet there" in a way. That would be too simple. (Interviewee 6)*
		"An online resource must be a supplement"<br><br>*Yes, I think you have to take the ... guidance first, and then in a way have it (the digital resource) as a... it might be a bit of extra information then... in the same way as showing them a brochure, «go in and look at this". Because there not always a need for you to look at it together /…/ yes... and so that it will be, in a way, a supplement to the conversation and the guidance we otherwise have. (Interviewee 1)*<br><br>*…but very ok that information is collected in a quality-assured place where we can safely say that "there you will find what we are talking about now. There you can read about what lies behind it or elaborate it more." (Interviewee 3)*<br><br>*No, it has to be something that we can, in a way, use in addition to what we have on the program, and which are communicating about. (Interviewee 4)*<br><br>*Sometimes there are mothers sitting here wondering about something - then I think "oh, now I should have a brochure", but we'd rather not use brochures anymore. I should have something to show off. "It is so difficult to*

		know what to make", they say. "What can we give them?" And then you try to come up with something. I certainly think it would have been nice to have something to show off. (interviewee 4)<br><br>*.. that we can give our information and guidance here, and that we can refer to and perhaps also use it in the consultation to some extent, but at least refer to (…) But that it can be a supplement and a place where parents themselves may search for information. And refer to that this is something they can do at home and talk to their partner about, because there are many times that only one of the parents comes to the child health center. Then such a tool would be useful to refer to. (Interviewee 6)*<br><br>*and in relation to that with... yes... overweight and such, it's not always easy to guide on that, or they are less receptive, and maybe if you show it here then... then they might look at it when they get home ... It's not always that it... is so well received, not everyone finds it easy to talk about it or accepting guidance ... but then ... if we can show a little and they themselves can sit at home and... maybe? (Interviewee 1)*<br><br>*And where it is vulnerable, those who might get a little angry when we talk about obesity issues... The way I see it, it might be a support for us... because then such a digital platform would be very neutral, whereas I would not be so neutral. So, then I could support the neutral information, I could build on that then, if I gave up the personal point of view, but referred to it, then maybe that would support the guidance. (Interviewee 3)*<br><br>*And our field of expertise is large, there is so much from the national guidelines that we have to address in our consultations. So for everything I put in, there must also be a professional assessment of what must then be taken out. So, it is up to us as a child's health service - if this should be included or if we have to show something - what should we take out then? (Interviewee 3)*<br><br>*And there is so much we have to talk about, and sometimes there are some really difficult issues... breakups and so on, there is so much that may come up like that. So, if it´s like "oh, I have to get that film in as well", it may be a bit... But then I think that, if necessary, if I see the need... (...) But getting it in to everyone, I think that can be difficult. (Interviewee 5)*

Theme II	Subthemes	
**The content should attract interest and be broad in scope**	***Advance essential and balanced knowledge on early nutrition***	*"Basic nutrition knowledge conveyed in an easy way"*<br><br>*In any case, there should be no room for misunderstandings. It is important to think about that. Maybe the use of language, it cannot be offensive ... I don't think that there are … that many things that aren't okay, really (...) but that there is some room for individual differences ... That it's not one answer to what to eat, there may be room for like … when the child doesn´t like something, he can try something else, right... (Interview 2)*<br><br>*And maybe, as I've been thinking about lately, try to make it less complicated. They don't have to go around being afraid of making mistakes, because there aren't that many mistakes to make. If you learn a few basic principles, then they you don't have to go around being so worried the first year. Because they are very much like that "oh, but how many days do I have to give this before I can give that?". It becomes like a dissertation on how they can start, rather than thinking intuitively. (Interviewee 3)*<br><br>*So, in some ways you have to find a balance where ... at least try to see what is good enough, and not give them worse conscience for everything that they can't afford, manage, or bear - and so on ... (Interviewee 4)*<br><br>*... and find a way that reach parents regardless of competence, dyslexia, language ... and the educational level of the parents. (…) That it should be suited for teenage parents as well as new, anxious parent at 40 years of age (…) It should somehow reach all without making them think that this is too simple or basic, and at the same time fit a 19-year-old. (Interviewee 3)*<br><br>*yes... they must in a way... want it and be interested and... some, we see that with the other websites we recommend… some are very keen, and others are just not interested. And some parents have an easier time figuring it out than others, so that (…) While maybe those who haven't … those who doesn't have many thoughts about it in a way, they just google it and then they find an answer, so these are the ones you in a way would like to reach then ... (Interviewee 5)*
		"Must be broad in content"<br><br>*Eh... so, there are often questions that you have to figure out, that you haven't thought about and such, but... there are a lot of questions anyway... the first, like when they start with food then, solid food. And... which food to serve the child? (Interviewee 1)*

		Yes, that assessment right here and now for each child when the nutrition is still breast milk, when is it right for my particular child to start with solid food in addition to milk? (…) And yes, I would like it to have information on that. Help and support for this part... (Interviewee 3)<br><br>*yes, maybe a bit of that... I feel that many people want the best for their children, but then they make mistakes because they don't know, for example, how many calories there are in... various products then ... i.e. everything that has a bit of hidden calories... like e.g. dairy butter or whole milk or yoghurt... ready-made food. The food that they really think it's healthy, but it's not... (…) And the amount of food... how much food should a child have? Because some parents are very satisfied if the child has eaten a bunch of slices of bread, but you don't need all that when you are two years old... so the amount of food and, it might be a good idea... to inform parents about that (Interviewee 2)*<br><br>*... then I think about having enough iron. A diet rich in iron then... so not additives, but what is found naturally in food, where to find it most easily and how important it is ... And the combination of calcium and vitamin D... (Interviewee 4)*<br><br>*Yes, many people have questions on allergies, particularly if they have allergies themselves, what to do then... if the child has reacted a bit to milk then... the first months, then ... when can we start introducing milk again? (...) and when they are a little bigger and, they still... "no, she can't drink milk ... we've taken all the milk away, and she´s so much better".. then you can... yes.. have some thoughts about it then... but they often have very strong opinions about it and then... then it might be a bit difficult to... discuss it or... (Interviewee 1)*<br><br>*yes, there are some children who are diagnosed early with milk allergy then. I feel ... I haven´t worked here that long, but I think that it has become more and more of it. And then the mother must eat a dairy-free diet... (…) Yes... so perhaps this is more frequently seen in the population, isn't there more irritable bowel disease and celiac disease and things like that? That ...you could include some of that ... (Interviewee 2)*<br><br>*And I have thought for many years that normalizing food and allergies in children... And if they get an allergy, they get it. Make food a normal thing ... it's not that dangerous, it's not that difficult, really. (Interviewee 3)*<br><br>*I don't know if it is included, but this with vegetarian or alternative diets. Or diets in general ... taking away foods that the parents think the child can't tolerate - we see it a lot. (…) Yes, too often the parents have a very... they are very determined that "My child can't eat this and that", and it is really just nonsense (Interviewee 5)*<br><br>*It is often the case that many parents have thoughts that the child cannot tolerate this or that. And there is no academic basis for saying so, but nevertheless they have made up their minds. So, a bit about such things would also*

		be useful, I think. But there's also something about not focusing too much on it, because then maybe even more people will think about it...but it's something I see more of now...intolerant to milk, intolerant to gluten or yes... (Interviewee 6)
	Promote food enjoyment and healthy eating	*Yes, I think that then... there are those who struggle, if the child is not fond of bread, for example, or you have a type of genetic variant, a bit of a difficult, picky eater type. There are a lot of children who, I see that often either the mother or father may have been like that as a child, right, and then it gets better around 6-7 years of age and then... maybe have a resource for this? (...) I think it would be quite... nice for the parents to see that "it's not just my child who is like that, this is a known problem". (Interviewee 2)*<br><br>*And I also think ... in the case of overweight or underweight for that matter, we have that too. Or with eating problems, like "Oh, it's just a mess with the food for this kid", then it would be nice to use it when needed. It could be good to have something like "Let's watch a movie together that has a lot of really good tips that you can work with" (Interviewee 5).*<br><br>*No, but what I think many parents can struggle with is that the children don't like the food, don't want to taste it and things like that. And they can easily fall into the trap of offering something else instead. So, I think it can be very useful for many to get advice, help and guidance on such issues. And provide support to have the strength and courage to stand in situations where the child does not want or wish to taste." (Interviewee 6)*<br><br>*… something that I think could be useful for the users? It's more support in the ... what should I say, the sensing of food. Not just physically getting food into the stomach, but sensing, learning about food, oral motor skills, enjoying the meal and the food. That this has to be a very big part of the package, that it's not just about which nutrients and which types of food (...) The early introduction of food, the sensing and the positive approach to nutrition ... (Interviewee 3)*<br><br>*Yes, yes, it's a big topic. And maybe a little bit about this ... who's in charge here? You're the adult and you decide what goes on the table and what's available in the cupboard (...) And the interplay between food and emotions, to use a soon-to-be worn-out expression. But it's so intertwined, so making it positive – that it (the resource) can help us to focus on it together with the parents. (Interviewee 5).*

Theme III	Subthemes	Sample quotes
**An online resource must be easy to use and adapted to different users**	***Be easy to navigate, visual and concrete***	*Yes, everyone has it now. Maybe not a PC at home, but smartphones have... almost 99,9% must have it, that's my impression. Because here you get a text message the day before "remember you have an appointment for little ..." or ... yes (Interviewee 2).*<br><br>*Yes, and the last time we talked about it on another occasion, the conclusion was that smartphones are the thing. Not a computer, because not everyone has a computer." (Interviewee 3)*<br><br>*Oh yes, everyone has (...) It... I've hardly met any families who don't have a smartphone. (...) No, they're very keen on it. Both to be able to Skype or have some Facetime conversations with family in their home country and... no, so everyone has one (Interviewee 4)*<br><br>*... but it must be easy to find your way around and so on... simple in that sense. Yes... (Interviewee 1)*<br><br>*No, I think it's just how you access the information. So, we have a website in the municipality and there are a lot of steps to take to enter it. And I can only show it to a few people who... well, those who can read a little Norwegian and can find their way around, and then I have to sort of write down all the steps. But there are a lot of things you have to click on to find the right information, so it's more about how easily accessible it is. (Interviewee 4)*<br><br>*It must at least be easy to find and said in a very simple way (Interviewee 5).*<br><br>*No... I think concrete and short, if there are a lot of explanations, then people don't bother to read." (Interviewee 2)*<br><br>*And those who have few resources, if there's a lot to read, they may drop out. (...) At least according to feedback on other things we've had over the years, things with a lot of text, people don't bother with that, but a bit like that ... point by point (...) Yes, I think at least in order to to have an effect on living conditions and such, that it has to be short and concise, so they can delve deeper if they want to, but I think many people drop out if you have to read to the bottom before yoy get the clue. (Interviewee 3)*<br><br>*so things have to be... they have to be... easy to use. And not least, I think visually... so that it's not just words and... mhm (...) Because we use the words ourselves. Use the visuals to a greater extent, and then those who are perhaps even more interested can click through to more concrete, theoretical information in a way (Interviewee 4).*<br><br>*It's certainly important that the information is easy to find and said in a very simple way, and maybe a bit like... So that it's not too boring, that it's a bit exciting, I was going to say. (...) Yes, make it short. (Interviewee 5)*

	Be adapted to different cultures and languages	*Yes, it would be nice if it were in many languages. Because it's often very challenging, especially perhaps those who... know a little Norwegian... who you might not have an interpreter for, because you think that this is fine, right... and then they might not want it either. And I've experienced that many times, when they come back... you think they understand, and then they don't. (...) (Interviewee 1)*<br><br>*It is perhaps typical that ... yes ... those who do not master Norwegian so well, then there must be different language variants available ... it will be easier. (...) Yes, not just English, there must be other languages as well (...) And then you must have room for other cultures here, not everyone is a nation of bread-eaters ... (Interviewee 2)*<br><br>*So otherwise, the big obstacle is language (...) Yes. If there is to be an offer a for everyone, it must of course be in different languages. We have many multilinguals ... and that's often where many of the social differences lie in the first place." (Interviewee 3)*<br><br>*Well... it depends on it being in a familiar language... because... It takes a long time before they can master that type of information (...) because there are details and nuances that are not... when you have to explain things or talk about things that... are a little deeper than simple "yes and no" and... yes... so it has to be... available in other languages. (Interviewee 4)*

Theme IV	Subthemes	Sample quotes
**Taking part in a development process of an online resource on early nutrition seems meaningful**	***Relevant to influence the development of a tool on a central theme***	*It's to get some ... input then, on what ... or that we can help decide a little about what it contains. And ... you might get a closer relationship with it too, when you've been involved, it's not just a new email with a new (...) No, but the fact that you get the opportunity to provide input. And maybe if we can do this together, that sounds nice (Interviewee 1).*<br><br>*I think it's good... yes... I'm in favor of it (...) I think so. Because food for those children is a recurring topic we often talk about." (Interviewee 2)*<br><br>*…but this one of our main topics, isn't it? And it has a preventive... long-term preventive effect, what we try to... what we can work on here. So... I definitely think so, and where we always seem to get stuck is how many projects we can fit into our everyday life ... well, all the topics we sort of... But this is a theme, a recurring theme. There's always breastfeeding, and there's always nutrition and there's always... yes... growth, well-being, development... (Interviewee 4)*<br><br>*So, diet is linked to many lifestyle diseases. In terms of prevention, it's very important (...) We're in a period of a lot of projects right now, and that's been my little concern in all of this (...) Because we feel like we're being inundated with things that we have to use and communicate and incorporate all the way. But if it's something short-term that we're involved in, and if it results in good material that we can use, then I think there's interest in that... (Interviewee 5)*<br><br>*Yes, I think so. I don't think that ... It doesn't have to mean that you spend so much more time on it, necessarily, so I think we'll manage to do that for the most part. (Interviewee 6)*
	Facilitates information sharing and a common knowledge base	*No, I think it can help to raise the profile of nutrition (Interviewee 5).*<br><br>*I think it's very exciting because we get to raise these topics again, and we get a bit of the latest research and things like that. And a bit more interdisciplinary in a way, so you learn more, which is very exciting. It's definitely positive for the child health centre to be involved in something like that (...) Yes, I think we're generally very interested in gaining more knowledge. I definitely think so (Interviewee 6).*<br>*... I haven't worked that long, others have worked for more than 30 years. But things change... all the time, and then there's something about us... that we all give the same advice... because it's a bit like that... we sit in our own offices and... yes, it's important... that we don't give advice in different ways, even though it has to be adapted to each individual family... (Interviewee 1)*

		... and that's how it is with all topics, to always ... aligning ourselves and being on the same level. Maybe it's easier to (...) Yeah, I don't know. Quality assurance for users, because we're very concerned about that. That what we deliver must have the same weight, or be at the same level. And that it should be the same, that it doesn't matter if they come to see me or her. (Interviewee 3).<br><br>*Because there's something about the fact that we have a very high goal of trying to give fairly similar advice. We don't quite manage to achieve this goal, because there are eleven different people who have to do it. We have the Directorate of Health's guidance as a kind of cornerstone, but there are still many different elements in it. So, I think we need to focus on that and maybe zoom in on ... or correct ourselves a little bit perhaps?" (Interviewee 5)*
